# Swallowability of Minitablets among Children Aged 6–23 Months: An Exploratory, Randomized Crossover Study

**DOI:** 10.3390/pharmaceutics14010198

**Published:** 2022-01-15

**Authors:** Nao Mitsui, Noriko Hida, Taro Kamiya, Taigi Yamazaki, Kazuki Miyazaki, Kiyomi Saito, Jumpei Saito, Akimasa Yamatani, Yoichi Ishikawa, Hidefumi Nakamura, Akihiro Nakamura, Tsutomu Harada

**Affiliations:** 1 Division of Pharmaceutics, Department of Pharmacology, Toxicology and Therapeutics, School of Pharmacy, Showa University, 1-5-8 Hatanodai, Shinagawa-ku, Tokyo 142-8555, Japan; n.suzuki@cmed.showa-u.ac.jp (N.M.); k-miyazaki@pharm.showa-u.ac.jp (K.M.); kiyomi-s@pharm.showa-u.ac.jp (K.S.); hironak@pharm.showa-u.ac.jp (A.N.); 2 Department of Pharmacology, Clinical Pharmacology School of Medicine, Showa University, 6-11-11 Kita-karasuyama, Setagaya-ku, Tokyo 157-8577, Japan; n.hida@med.showa-u.ac.jp; 3 Clinical Research Institute for Clinical Pharmacology and Therapeutics, Showa University Karasuyama Hospital, 6-11-11 Kita-karasuyama, Setagaya-ku, Tokyo 157-8577, Japan; t.yamazaki@cmed.showa-u.ac.jp; 4 Department of Pediatrics, School of Medicine, Showa University, 1-5-8 Hatanodai, Shinagawa-ku, Tokyo 142-8555, Japan; kamiya-t@med.showa-u.ac.jp; 5 Department of Pharmacy, National Center for Child Health and Development, 2-10-1 Okura, Setagaya-ku, Tokyo 157-8435, Japan; saito-jn@ncchd.go.jp (J.S.); yamatani-a@ncchd.go.jp (A.Y.); 6 Department of Pediatric Medication, Meiji Pharmaceutical University, 2-522-1 Noshio, Kiyose-shi, Tokyo 204-8588, Japan; ishikawa@my-pharm.ac.jp; 7 Department of Research and Development Supervision, National Center for Child Health and Development, 2-10-1 Okura, Setagaya-ku, Tokyo 157-8435, Japan; nakamura-hd@ncchd.go.jp

**Keywords:** fine granules, liquid formulations, infants

## Abstract

Minitablets have garnered interest as a new paediatric formulation that is easier to swallow than liquid formulations. In Japan, besides the latter, fine granules are frequently used for children. We examined the swallowability of multiple drug-free minitablets and compared it with that of fine granules and liquid formulations in 40 children of two age groups (*n* = 20 each, aged 6–11 and 12–23 months). We compared the percentage of children who could swallow minitablets without chewing with that of children who could swallow fine granules or liquid formulations without leftover. The children who visited the paediatric department of Showa University Hospital were enrolled. Their caregivers were allowed to choose the administration method. In total, 37 out of 40 caregivers dispersed the fine granules in water. Significantly more children (80%, 95% CI: 56–94%) aged 6–11 months could swallow the minitablets than those who could swallow all the dispersed fine granules and liquid formulations (22%, 95% CI: 6–47% and 35%, 95% CI: 15–59%, respectively). No significant differences were observed in children aged 12–23 months. Hence, minitablets may be easier to swallow than dispersed fine granules and liquid formulations in children aged 6–11 months.

## 1. Introduction

In 2000, the International Council for Harmonisation of Technical Requirements for the Registration of Pharmaceuticals for Human Use (ICH) enacted the “Guideline on Clinical Investigation of Medicinal Products in the Paediatric Population” (ICH-E11) [1], which states that specific paediatric formulations are required for paediatric patients. Paediatric formulations need to be age-appropriate, have an acceptable taste and size, and be easy to use by the caregivers [2]. The World Health Organization (WHO) recommends using oral dosage forms for paediatric patients [3]. In contrast, since the per capita dose of paediatric oral formulations is lower than that of adults and the market size is small, pharmaceutical companies need to exercise economic rationality in development and manufacturing. Hence, the development of globally accepted and highly versatile paediatric formulations is needed.

Amidst such social demands, in contrast with syrup, which is commonly used in Europe, tablets with a diameter of 2–4 mm, which are called minitablets [4], have been garnering interest as formulations with excellent stability as well as antiseptic and antifungal properties. Minitablets have potential applications as a flexible drug delivery tool in addition to their generally perceived use as multi-particulates [5]. Additionally, for combination therapies where multiple active ingredients are simultaneously dosed, the use of minitablets will enable independent adjustment of each dose [6]. Moreover, their unpleasant taste can be masked by coating their surface. In 2009, Thomson et al. divided a cohort of children aged 2–6 years into four age groups and tested whether they could swallow one minitablet with a diameter of 3 mm. This study demonstrated the potential to use minitablets for the treatment of preschool-aged children and suggests that minitablets can be used as a potential new formulation for children in this age range [7]. Following this study, in 2012, Klingmann et al. assigned children aged 6 months to 6 years to six age groups. The swallowability of the minitablets with a diameter of 2 mm was compared to that of 3 mL of syrup. The percentage of children who were able to swallow a minitablet without chewing (52.9–88.2%) was significantly higher than that of children who were able to swallow the syrup without any leftover liquid in their mouth [8]. These results suggest that minitablets may be a new paediatric alternative formulation to syrup.

Since only a small amount of the active ingredient can be contained in one tablet, patients would need to take multiple tablets if these were used in clinical practice. Kluck et al. reported that more than half of the children aged between 2 to 3 years could safely swallow up to 10 minitablets when jelly foods were used during the administration [9]. In 2018, the swallowability of multiple minitablets was compared with that of syrup [10]. The results revealed that the swallowability of the minitablets was not superior to that of the syrup in the 2–5-year-old children; however, the percentage of children aged 6 months to 2 years who were able to swallow 25 and 100 minitablets without chewing was 80% and 75%, respectively. These percentages were significantly higher than that of children of the same age who were able to swallow syrup without liquid leftover.

In Japan, where the methods of administration used are different from those employed in Europe, fine granules are frequently used in addition to liquid formulations. However, no studies comparing the swallowability of these formulations with that of minitablets have been published [11]. Thereby, in 2019, we divided children aged 2–8 years into three age groups and compared the swallowability of multiple minitablets (2–4 years old: 6 tablets; 4–7 years old: 9 tablets; 7–8 years old: 12 tablets) with that of fine granules and liquid formulations [12]. The results revealed that 71% of the children were able to swallow the minitablets without chewing, which was a significantly lower percentage than that of children who were able to swallow fine granules and liquid formulations without leftover. This may be due to the fact that the percentage of children who chewed the mini tablets increased with decreasing age. Particularly, approximately 60% of children aged 2–4 years chewed the minitablets.

To improve children’s adherence to medication, minitablets should be swallowed without chewing to ensure that children do not taste the active ingredient in the minitablets. However, when taking one minitablet, 30% of the children aged 2–4 years chewed the minitablet [7] and 25–30% of children aged 6 months to 4 years chewed the minitablet [8]. In contrast, Klingmann reported that 15–30% of children aged 2–5 years who were administered 100 minitablets chewed them, while only approximately 10–20% of children aged 6 months to 2 years did so [10]. These studies suggest that children under the age of 2 are more likely to swallow minitablets without chewing. Therefore, we conducted an exploratory study on the swallowability of minitablets, fine granules and liquid in children 6 months to 2 years old.

In previous European studies, Thomson et al. gave explicit minitablet administration instructions to caregivers [7], and Klingmann et al. administered the minitablets themselves to the children [8,10]. However, since the caregivers are the ones that usually administer the drug to their infants, ideally, the easiest administration method should be chosen for each caregiver. Nales et al. reported that caregivers of children of 1 to 4 years old preferred minitablets better than powder or suspension [13]. No other studies comparing the ease of use and/or the preference of minitablets with that of other formulations from the perspective of the caregiver have been published. Therefore, we let the caregivers decide the administration method for themselves and surveyed the caregivers after drug administration regarding the ease of use of the formulation chosen, and their preference to use it in the future using a questionnaire.

## 2. Materials and Methods

### 2.1. Ethics Approval

This study was planned as a prospective, randomised, open-label, three-group crossover study at Showa University Hospital and was performed in accordance with the principles of the Declaration of Helsinki. This study was conducted from December 2020 to February 2021 based on a protocol (UMIN000042559) approved by the Institutional Review Board of the Showa University School of Medicine (Approval No. 3276).

### 2.2. Subjects

The subjects were children aged 6–23 months (6–11 months: 10 boys and 10 girls; 12–23 months: 10 boys and 10 girls) who were hospitalized or received outpatient treatment at the Showa University Hospital. Children who had deglutition issues or a history of drug allergies were excluded from the study. Since the subjects were not able to make decisions on their own, we explained the study contents to their caregivers in writing and asked the caregivers to provide their written consent.

### 2.3. Description of the Three Formulations

In this study, we used three types of formulations: minitablets, fine granules, and liquid formulations. The minitablets comprised 95% D-mannitol (Merck KGaA, Frankfurt, Germany) and 5% magnesium stearate (Taihei Chemical Industrial Co., Ltd., Tokyo, Japan). They were manufactured at the GMP-compliant Paediatric Formulation Laboratory of National Center for Child Health and Development. The minitablets were cylindrical, had a diameter of 2 mm, a thickness of approximately 2 mm, and weighed approximately 10 mg. Their hardness was approximately 2.2 ± 0.20 kgf (*n* = 5), and their disintegration time in water was 3 min 21 s to 5 min 46 s (*n* = 6). The fine granules comprised 86% D-mannitol (Mitsubishi Corporation Food Tech, Tokyo, Japan), 3% hydroxypropyl cellulose (Nippon Soda, Osaka, Japan), 10% corn starch (Nihon Shokuhin Kako, Okayama, Japan), and 1% light anhydrous silicic acid (Taihei Chemical Industrial Co., Ltd., Tokyo, Japan). They had a median diameter D50 of 263.2 ± 107.8 μm and were manufactured by Sawai Pharmaceutical Co., Ltd., Osaka, Japan. A 10% sucrose solution was used as the liquid formulation by diluting the simple syrup (85% sucrose solution) listed in the Japanese Pharmacopoeia with drinking water. The sweetness of a 10% sucrose solution is almost equal to that of the 15% glucose syrup used in our previous study. Moreover, the viscosity of the liquid formulation was 1.35 ± 0.07 mPa·s (*n* = 3). Assuming that the formulation was cefditoren pivoxil (3 mg (titre)/kg), which is frequently administered to children, the amount of each formulation to be administered was calculated based on the average weight of a typical child (6–11 months: 8 kg; 12–23 months: 10 kg). The active ingredient content was assumed to be 60% per minitablet and 10% for the fine granules. Table 1 shows the dosage of each formulation for each age group.

### 2.4. Administration Procedure

**Figure 1:**
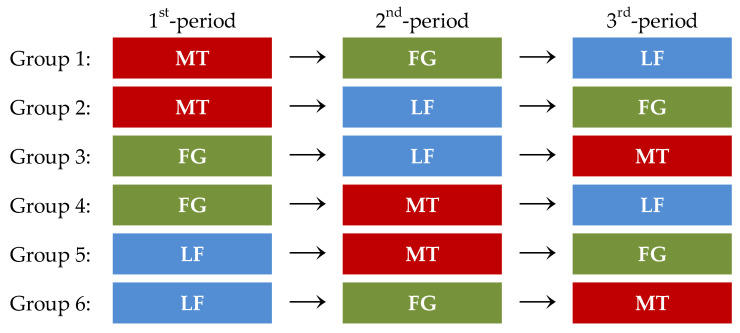
Administration sequence of the minitablets (MT), fine granules (FG), and liquid formulations (LF).

The age of the subjects was considered as a stratification factor (6–11 months and 12–23 months). The subjects were randomly assigned to six groups in the order of consent acquisition to eliminate the influence of the dosing sequence (Figure 1). In an isolated and quiet room of the hospital, the caregivers administered each formulation to their infants according to the dosing sequence predetermined for each group. The caregivers were free to choose the administration method, and a dropper, spoon, cup, and drinking water were available for them to use freely. Considering the burden imposed on the subjects, the administration had to be performed within 15 min.

### 2.5. Evaluation

The primary evaluation was based on the observations made by one healthcare professional, and the swallowability of the formulation was evaluated according to the five criteria shown in Table 2. The criteria for fine granules were added to the criteria used in the previous study [8,10,12]. The administration method by caregivers was carefully observed.

As a secondary evaluation, after the administration of all the formulations, a survey in which caregivers ranked each formulation in terms of ease of use and intention to use in the future was conducted.

### 2.6. Statistical Analyses

All significance levels were set to 5%. All statistical analyses were performed with EZR (Saitama Medical Centre, Jichi Medical University, Saitama, Japan), which is a graphical user interface for R (The R Foundation for Statistical Computing, Vienna, Austria). More precisely, it is a modified version of R commander designed to add statistical functions frequently used in biostatistics [14]. For the analysis of the primary evaluation, the percentage of observations that followed criterion 1 for the minitablets, fine granules, and liquid formulations and the 95% confidence intervals were calculated for each age group (6–11 months and 12–23 months). Moreover, these formulation percentages were compared using McNemar’s test.

## 3. Results

Most caregivers added water to the fine granules and administered them to the children as dispersed fine granules (DFG). In other words, 18 out of the 20 children aged 6–11 months and 19 out of the 20 children aged 12–23 months ingested powdered fine granules as dispersed fine granules in water. Therefore, we decided to compare the swallowability of the minitablets and liquid formulations with that of dispersed fine granules in water, instead of with that of powdered fine granules.

### 3.1. Criteria 1 (Swallowed)

**Figure 2:**
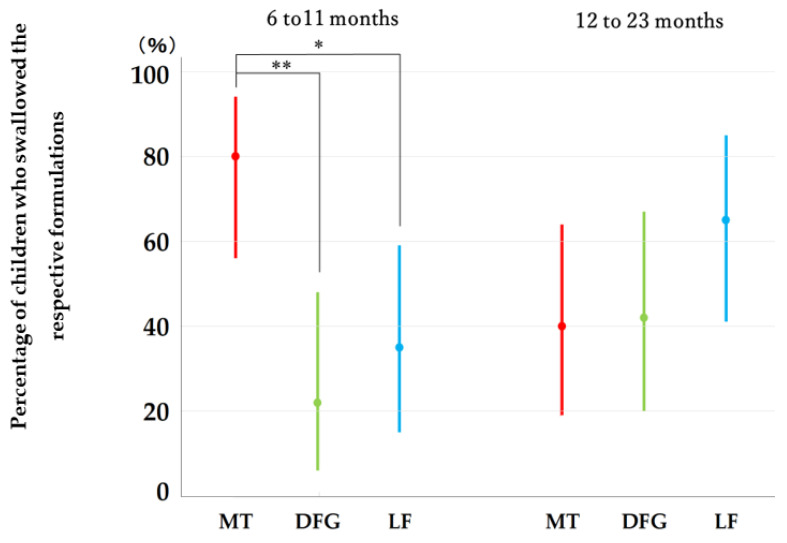
Percentage of swallowed minitablets (MT), fine granules (FG), and liquid formulations (LF). Each bar represents the 95% CI. McNemar’s test, * *p* < 0.05, ** *p* < 0.01.

Figure 2 shows the percentage of children who were able to swallow the minitablets, dispersed fine granules, and liquid formulations without leftover. In the group comprising children aged 6–11 months, 80% or 16 out of 20 children were able to swallow four minitablets without chewing (95% Cl: 56–94%), 22% or four out of 18 children were able to swallow the dispersed fine granules without leftover (95% Cl: 6–47%), and 35% or seven out of 20 children were able to swallow the liquid formulations without leftover (95% CI: 15–59%). The percentage of children who were able to swallow all minitablets without leftover was significantly higher than that of children who were able to swallow the dispersed fine granules and liquid formulations without leftover.

In the 12–23-month age group, 40% or eight out of 20 children were able to swallow five minitablets without chewing (95% CI: 19–64%), 42% or eight out of 19 children were able to swallow dispersed fine granules without leftover (95% CI: 20–67%), and 65% or 13 out of 20 infants were able to swallow the liquid formulations without leftover (95% CI: 41–85%). No significant difference was observed between these percentages for the three formulations.

### 3.2. Criteria 2 (Chewed or Small Leftover)

In the 6–11-month age group, no subject was able to swallow all four minitablets without chewing. A total of seven out of 18 children and seven out of 20 children were able to swallow the dispersed fine granules and liquid formulations, respectively, with a small amount leftover. In contrast, four out of 20 children aged 12–23 months chewed and swallowed all 5 minitablets. A total of two out of 19 children and four out of 20 children swallowed the dispersed fine granules and the liquid formulations, respectively, with a small amount leftover.

### 3.3. Criteria 3 (Spat Out), Criteria 4 (Inhaled/Coughed), and Criteria 5 (Refused to Take)

In the 6–11-month age group, one out of 20 children spat out the minitablets. One in 18 children spat out the dispersed fine granules, two coughed, and one refused to take them. One out of 20 children spat out the liquid formulations. In the 12–23-month age group, two out of 20 children spat out the minitablets, and one child refused to take them. One out of 19 children spat out the dispersed fine granules, and one child coughed. One in 20 children coughed when administered the liquid formulations, and two children refused to take them.

### 3.4. Subjects Who Did Not Meet the Criteria (Children Who Quit the Study)

As for the children who did not meet the criteria of this study, in the 6–11-month age group, one subject was able to ingest three of four minitablets without chewing, and two children were able to ingest two minitablets without chewing. Three children refused to take the dispersed fine granules partway through the study. Four subjects took the liquid formulations but refused to take them partway through the study. In the 12–23-month age group, one, one, one, and two children ingested four of the five minitablets, three, two, and one minitablet, respectively, by chewing. Seven children were able to take the dispersed fine granules at first but refused to drink the suspension partway through, leaving about half of it (Supplementary Table S1).

### 3.5. Minitablet Administration Method and Swallowability

By observing how caregivers administered the minitablets to the children, we found that repeated administration of one minitablet at a time was the most commonly used method, being utilized in 16 patients in the 6–11-month group and 15 patients in the 12–23-month group. Two individuals administered the minitablets by breaking them into multiple pieces in both age groups. Two caregivers administered four minitablets at a time to children aged 6–11 months, and three caregivers simultaneously administered five minitablets to children 12–23 months. When the minitablets were administered one by one, 13 out of 16 (81%) children aged 6–11 months and five out of 15 (33%) children aged 12–23 months swallowed all tablets without chewing. Furthermore, 100% (16 out of 16) and 87% (13 out of 15) of the children swallowed the first minitablet without chewing in the 6–11-month and 12–23-month age groups, respectively. Among the children who took multiple doses of four minitablets, only one child (6–11 months) was able to swallow all of them without chewing. All five children who were administered all at once, swallowed them without chewing.

### 3.6. Questionnaire Survey of Caregivers

For the secondary evaluation, the rankings of the formulations in terms of ease of use by the caregivers and the products they would like to use in the future were tabulated for each formulation. Table 3 shows the ranking of formulations based on the ease of use, while Table 4 shows the ranking of formulations based on the caregivers’ intention to use them in the future. The minitablets were the easiest formulation to use in the 6–11-month group, and the liquid formulations were the easiest to administer in the 12–23-month group. In both the 6–11-month group and the 12–23-month group, caregivers said that they intended to use minitablets in the future. None of the caregivers ranked the dispersed fine granules as the best formulation in terms of the ease of use and intention to use them in the future.

## 4. Discussion

To date, fine granules and liquid formulation are often prescribed to children in Japan, and minitablets are not commercially available. The present is the first study conducted in Japan involving the administration of minitablets to children aged 6–23 months.

This study focused on whether children could swallow all minitablets without chewing. We found that 80% of the children aged 6–11 months were able to swallow all four minitablets without chewing. In contrast, 40% of the children aged 12–23 months were able to swallow all five minitablets without chewing. These results were comparable to those obtained in the previous study by Klingmann et al. [8], which may be explained by the fact that children start to grow molars at around one year of age. Children aged 6–11 months have no molars and cannot chew minitablets. Furthermore, since pre-weaned children swallow food via infantile swallowing, which involves a sucking motion [15], they may be able to swallow minitablets smoothly. In future studies, it seems necessary to investigate not only the age of children, but also the developmental stages of deglutition, such as the stage of weaning and the presence of deciduous teeth.

In our previous study on children aged 2–8 years [12], we found that 33.3% of children aged 2–4 years were able to swallow all six minitablets without chewing. These values were lower than the percentage of children aged 6–11 months and that of those aged 12–23 months that were observed to swallow the minitablets in this study (80% and 40%, respectively). These results are similar to those of the study by Klingmann et al. reported: only around 30% of children aged 2–5 years were able to swallow all of the minitablets before chewing them. In comparison, approximately 75% of children aged 6 months to 2 years could do the same, when 100 tablets were administered [10]. These studies suggested that it may be difficult for infants and children to swallow minitablets without chewing at around 2 years of age due to various morphological and functional changes associated with the development. In terms of safety, no adverse events were observed in children who took minitablets in this or previous studies conducted in Europe. Münch et al. reported that one oblong tablet (2.5 × 6 mm), which is larger than minitablets of 2 mm, can be taken by children aged 1 to 5 years as a safe alternative to liquid formulations [16]. This suggests that minitablets with a diameter of 2 mm can be administered safely to Japanese children aged 6 months and older.

In this study, no specific instructions were provided for the administration of minitablets, and approximately 80% of caregivers administered the minitablets one by one. This may have been a result of caution exercised by the caregiver as this was the first time that they administered minitablets to their children. A total of 100% of children aged 6–11 months and 87% of children aged 12–23 months who were administered the minitablets one by one were able to swallow the first minitablet without chewing. However, 81% of children aged 6–11 months and 33% of children aged 12–23 months were able to swallow all 4 or 5 minitablets without chewing. This result suggests that when minitablets are repeatedly administered within a short period, children aged 6–23 months may find it difficult to take the tablets. In contrast, all five children who were given the minitablets at once were able to swallow them without chewing. Administering multiple minitablets at once may make it easier to swallow them. Regarding the swallowing of multiple minitablets at once, Klingmann et al. reported that approximately 80% of children aged 6 months to 2 years were able to swallow 25 tablets at once without chewing, and approximately 75% of children were able to swallow 100 tablets [10]. No adverse events, such as choking or coughing, were reported. In further study in Japan, it may be better to advise caregivers to administer multiple minitablets all at once.

As reported by Saito et al. [17], parents and nurses often dissolve or disperse powder formulations in water for children. Alessandrini et al. also reported that liquid formulations were widely selected by children less than 12 years and granules were not appreciated, particularly by adolescents [18]. In this study, many parents also dispersed the fine granules in water and administered them. In the 6–11-month age group, the percentage of children who were able to swallow minitablets without chewing was higher than dispersed fine granules or liquid formulations. It may be easier for children aged 6–11 months to swallow minitablets than fine granule dispersions or liquid formulations. The percentage of infants who were able to swallow these two liquid types was low indicating that the liquid preparations are difficult to administer to infants. Part of the reason why children had difficulty in swallowing the dispersed fine granules was that the corn starch (10%) and the light anhydrous silicic acid (1%) in the dispersed fine granules are almost insoluble in water, and D-mannitol (86%) is only partially soluble in the first several seconds. As such, the mouth feeling induced by the dispersion may have affected the swallowability of the dispersed fine granules. Therefore, when developing fine granules that may be administered as a dispersion, it may be necessary to consider their texture, such as its roughness in the oral cavity, and devise measures.

After the study, the formulations were ranked by them based on the ease of use. Caregivers reported that the liquid formulations were the easiest to use. In this study, the researchers weighed the dose of the liquid formulations in a cup and handed it to the caregivers. As such, the preference of the caregivers toward liquid formulations may be because they required the least amount of time and effort to administer. Many caregivers chose to administer the minitablets one by one, which made the administration bothersome and longer. Nevertheless, it is worth noting that the number of caregivers who ranked minitablets in the first place was higher than those who ranked liquid formulations. In contrast, no caregivers ranked fine granules in the first place, which may be attributed to the fact that it took more time and effort to disperse the fine granules in water. Nales et al. also reported that the caregivers preferred the minitablets or syrup over the powder or suspension [9]. In this study, many caregivers ranked the minitablets in the first place as a formulation that they intend to use in the future. Seven caregivers chose the liquid formulation as the easiest formulation to use; however, they also selected the minitablets as a formulation that they would want to use in the future. Some caregivers thought that the minitablets were a little harder to manipulate than the liquid formulations, probably because this was their first time using the former. Based on this information, we feel that the caregivers prefer the development of easy-to-use minitablets.

This study has a few limitations. Since this was an exploratory study, these results alone are not enough to determine the ideal formulation for infants. Furthermore, the criteria used in this study were established for a single-dose administration; however, many caregivers chose to administer minitablets one by one. We may have to modify the evaluation method considering the experience in this study. As the number of subjects was small and only included children aged 6 months and older, it is necessary to conduct a study with a larger cohort that includes children younger than 6-month-old.

## 5. Conclusions

In Japan, fine granules and liquid formulations are frequently used for children. In light of this, we conducted an exploratory study to examine the swallowability of minitablets as a new oral formulation for children, as well as to examine its ease of use by the caregivers. The results revealed that children aged 6–23 months can take minitablets without adverse events. Furthermore, 80% of the children aged 6–11 months were able to swallow all four tablets without chewing, suggesting that it may be easier to take these than to take dispersed fine granules or liquid formulations. Moreover, many caregivers found minitablets easy to use and expressed the intent of using them in the future. Therefore, we believe that minitablets, which are currently being developed, mainly in Europe, are a viable option for children in Japan. We believe that this study contributes to developing an easy-to-take minitablet formulation for children.

## Figures and Tables

**Table 1:** Dosage of minitablets (MT), fine granules (FG), and liquid formulations (LF) for each age group.

Age (in Months)	MT (Tablets)	FG (mg)	LF (mL)
6 to 11	4	240	3
12 to 23	5	300	3

**Table 2:** The evaluation criteria used for the swallowability outcome of minitablets (MT), fine granules (FG), or liquid formulations (LF).

Criteria	MT	FG	LF
**1**	**Swallowed:** No chewing during deglutition and no solid residuals found during the oral inspection	**Everything swallowed:** No granules/suspension out of the mouth before or during deglutition	**Everything swallowed:** No liquid trickling out of the mouth before or during deglutition
**2**	**Chewed:** Chewing was observed before deglutition and/or whole solids or parts of the solids were found during the oral inspection	**Small leftover:** Some granules/suspension trickling out of the mouth or leftover present in mouth or spoon	**Small leftover:** Some liquid trickling out of the mouth or leftover in the mouth or spoon
**3**	**Spat out:** No deglutition was observed; the solid was spat out of the mouth of the child	**Spat out:** No deglutition was observed because the child expelled the granules/suspension	**Spat out:** No deglutition was observed because the child expelled the liquid
**4**	**Inhaled/coughed:** Minitablets were inhaled or induced cough during deglutition	**Inhaled/coughed:** Parts of the granules/suspension were inhaled or induced cough during deglutition	**Inhaled/coughed:** Parts of the liquid were inhaled or induced cough during deglutition
**5**	**Refused to take:** The child did not allow the caregiver to place the solid in their mouth	**Refused to take:** The child did not allow the caregiver to place the dropper/medicine spoon in their mouth	**Refused to take:** The child did not allow the caregiver to place the dropper/medicine spoon in their mouth

**Table 3:** Ranking of the formulations based on the ease of use by the caregivers.

Formulation	Caregivers of Children Aged 6–11 Months (*n* = 18 *)	Caregivers of Children Aged 12–23 Months (*n* = 19 **)
MT	1st: 9	1st: 8
	2nd: 5	2nd: 6
	3rd: 4	3rd: 5
DFG	1st: 0	1st: 0
	2nd: 4	2nd: 7
	3rd: 14	3rd: 12
LF	1st: 9	1st: 11
	2nd: 9	2nd: 6
	3rd: 0	3rd: 2

* Except for two caregivers (who administered FG in powder form), ** Except for one person (who administered FG in powder form).

**Table 4:** Ranking of formulations by the caregivers based on their intention to use them in the future.

Formulation	Caregivers of Children Aged 6–11 Months (*n* = 18 *)	Caregivers of Children Aged 12–23 Months (*n* = 19 **)
MT	1st: 14	1st: 10
	2nd: 2	2nd: 4
	3rd: 2	3rd: 5
DFG	1st: 0	1st: 0
	2nd: 3	2nd: 7
	3rd: 16	3rd: 12
LF	1st: 4	1st: 9
	2nd: 14	2nd: 8
	3rd: 0	3rd: 2

* Except for two persons (who administered FG in powder form), ** Except for one person (who administered FG in powder form).

## Data Availability

Data is contained within the article and supplementary material. Table S1. Observation results of the administration of formulations and the number of subjects who did not meet the evaluation criteria (Table 2).

## Supplementary Materials

The following are available online at https://www.mdpi.com/article/10.3390/pharmaceutics14010198/s1, Table S1: Observation results of the administration of formulations and the number of subjects who did not meet the evaluation criteria.
