# Supplementary material for: Swallowability of Minitablets among Children Aged 6–23 Months: An Exploratory, Randomized Crossover Study
Source: Pharmaceutics. 2022 Jan 15;14(1):198. doi: 10.3390/pharmaceutics14010198 (PMC8779905; doi:10.3390/pharmaceutics14010198)
Supplement: Supplementary file 1 [file pharmaceutics-14-00198-s001.zip › pharmaceutics-1531391-supplementary.pdf]

pharmaceutics

MDPI

Supplementary Materials: Swallowability of Minitablets among Children Aged 6–23 Months: An Exploratory, Randomized Crossover Study

Nao Mitsui, Noriko Hida, Taro Kamiya, Taigi Yamazaki, Kazuki Miyazaki, Kiyomi Saito, Jumpei Saito, Akimasa Yamatani, Yoichi Ishikawa, Hidefumi Nakamura, Akihiro Nakamura and Tsutomu Harada

Table S1. Observation results of the administration of formulations and the number of subjects who did not meet the evaluation criteria.

**Observation results of taking the formulations** **[How to take them]**	**No. of subjects**
**Minitablets** for children aged **6–11 months**	
The subject was able to swallow up to 3 minitablets without chewing but spat out the remaining tablet. [Subject repeatedly taking one minitablet at a time.]	1
The subject was able to swallow up to 2 tablets without chewing but then refused to take them; two tablets remained. [Subject repeatedly taking one minitablet at a time.]	2
The subject put the tablets in the mouth, but the tablets got caught between gums and lips; the subject then refused to take all the tablets. [Subject trying to take more than one tablet at a time.]	1
**Minitablets for children aged 12–23 months**	
The subject was able to swallow up to 4 minitablets by chewing, but then he spat out the remaining tablet. [Subject repeatedly taking one minitablet at a time.]	1
The subject was able to swallow up to 3 minitablets without chewing, but then he spat out one tablet and dropped the other. [Subject repeatedly taking one minitablet at a time.]	1
The subject was able to swallow up to 2 tablets without chewing but then refused to take them, and three tablets remained. [Subject repeatedly taking one minitablet at a time.]	1
The subject was able to swallow up to 1 tablet without chewing but then refused to take them, and four tablets remained. [Subjects repeatedly taking one minitablet at a time.]	2
**Fine granules for children aged 6–11 months**	
The subject put dispersed fine granules into the mouth but spat them out immediately. [Caregivers added water to the fine granules and administered them.]	1
The subject swallowed some of the dispersed fine granules but refused to take them. [Caregivers added water to the fine granules and administered them.]	2
The subject swallowed half of the dispersed fine granules but spat out the other half. [Caregivers added water to the fine granules and administered them.]	1
The subject put fine granules in the mouth but then spat out them. (Criteria 3) [Caregivers administered fine granules in powder form.]	1
The subject refused to put the fine granules in the mouth. (Criteria 5) [Caregivers administered fine granules in powder form.]	1
**Fine granules for children aged 12–23 months**	
The subject swallowed half of the dispersed fine granules, but then spat out the other half. [Caregivers added water to the fine granules and administered them.]	2

The subject swallowed some of the dispersed fine granules but refused to take them.<br>[Caregivers added water to the fine granules and administered them.]	3
The subject swallowed half of the dispersed fine granules, but dispersed powder and liquid remained.<br>[Caregivers added water to the fine granules and administered them.]	2
The subject swallowed some fine granules trickling a part out of the mouth. (Criteria 2)<br>[Caregivers administered fine granules in powder form.]	1
**Liquid formulations for children aged 6–11 months**	
The subject swallowed half of liquid formulations, but then spat out the other half.	1
The subject swallowed some of liquid formulations, but then refused to take them.	2
The subject swallowed part of liquid formulation, but a little remained in the dropper.	1
